# Computation and convergence of fixed-point with an RLC-electric circuit model in an extended b-suprametric space

**DOI:** 10.1038/s41598-024-59859-x

**Published:** 2024-04-25

**Authors:** Sumati Kumari Panda, Vijayakumar Velusamy, Ilyas Khan, Shafiullah Niazai

**Affiliations:** 1 Department of Mathematics, GMR Institute of Technology, Rajam, Andhra Pradesh 532127 India; 2 Department of Mathematics, School of Advanced Sciences, Vellore Institute of Technology, Vellore, Tamil Nadu 632 014 India; 3 Department of Mathematics, College of Science Al-Zulfi, Majmaah University, Al-Majmaah, 11952 Saudi Arabia; 4 Department of Mathematics, Education Faculty, Laghman University, Mehtarlam City, 2701 Laghman Afghanistan

**Keywords:** Applied mathematics, Pure mathematics

## Abstract

This article establishes various fixed-point results and introduces the idea of an extended b-suprametric space. We also give several applications pertaining to the existence and uniqueness of the solution to the equations concerning RLC electric circuits. At the end of the article, a few open questions are posed concerning the distortion of Chua’s circuit and the formulation of the Lagrangian for Chua’s circuit.

## Background summary and preliminaries

Numerous mathematical challenges incorporating the use of differential equations, and integral equations can be solved, and their continual existence is confirmed by the well-known contraction principle. To increase this extraordinary principle’s feasibility in numerous different abstract spaces, initiatives are being undertaken to explore and extend it^1–4^. By diminishing the triangle inequality, Bakhtin^5^ and Czerwik^6^ extended the configuration of metric space and called it b-metric space. As a result, a number of papers addressing fixed-point hypotheses for both single- and multi-valued mapping in b-metric space have been published.

Within this framework, Maher Berzig^7^ presented the idea of *b*-suprametric space by weakening the triangle inequality even further, while Kamran et al.^8^ presented the idea of extended *b*-metric space. A great deal of attention and study in this pursuit has been generated by the recent achievement of developing several extended/modified structures in metric spaces and/or their associated results (see,^9–18^).

RLC circuits, comprising resistors (R), inductors (L), and capacitors (C), are fundamental components in electrical engineering and electronics. These circuits play a pivotal role in various applications, including signal processing, power distribution, and filtering. In an RLC circuit, the behavior is governed by the interplay of these three passive components, each contributing distinct characteristics. Resistors dissipate energy in the form of heat, providing damping in the circuit. Inductors store energy in a magnetic field when current flows through them, resisting changes in current. Capacitors store energy in an electric field, resisting changes in voltage. Together, they create a complex interconnection of energy storage and dissipation mechanisms, giving rise to a rich array of circuit behaviors. RLC circuits exhibit phenomena such as resonance, where energy transfer between components reaches its peak efficiency, and transient response, where the circuit’s behavior changes over time in response to sudden changes in input. Understanding and analyzing RLC circuits are essential skills for electrical engineers, enabling the design and optimization of circuits for various applications in electrical and electronic systems. These components interact through the governing equations of Kirchhoff’s laws, leading to differential equations that describe the circuit’s behavior. RLC circuits find applications in various fields, from signal processing and filtering to power distribution and electronic devices, underscoring their significance in modern technology and engineering endeavors (see,^19–22^).

The analysis of an RLC circuit typically involves solving a system of linear differential equations rather than nonlinear integral equations. However, nonlinear integral equations can arise in certain special cases or when considering more complex circuit elements or behaviors. One scenario where a nonlinear integral equation may arise is when dealing with nonlinear elements such as diodes or transistors within the circuit. In these cases, the behavior of the circuit may not be describable solely through linear differential equations, and integral equations might be needed to model the relationship between voltage and current.

Many mathematical science difficulties, such as integral mathematical problems, can be addressed by restructuring other mathematical problems. Thus, the study of integral equations and the techniques for solving them are quite beneficial. Integral equations are used in the domains of science and engineering these days. Numerous scholars have devised several reliable techniques to deal with integral equations. One technique used by Rahman^23^ to solve an integral problem is the Chebyshev polynomials. When evaluating definite integrals, hybrid quantification has a greater level of accuracy, which leads to quicker convergence. For individual parameters, the linear symmetric formulation of Gaussian-Newtonian kind rules of less accuracy has been used to produce a combined quantification with improved accuracy. Both finite-element methods and computational solutions of integral equations have recently found effectiveness using combined quantification.

We propose the notion of an extended *b*-suprametric space in the below-stated formulation.

### Definition 1.1

Assume that ${\mathscr {M}}$ be a non-empty set also consider a function $\gamma :{\mathscr {M}}\times {\mathscr {M}}\rightarrow [1,\infty )$, and $b\ge 1$. A function ${\mathcal {E}}_{s}:{\mathscr {M}}\times {\mathscr {M}}\rightarrow {\mathbb {R}}^{+}$ is said to be an extended *b*-suprametric if for all $\vartheta ,\varrho ,\omega \in {\mathscr {M}}$ the following properties hold:

1. $${\mathcal {E}}_{s}(\vartheta ,\varrho )=0 \ \text {iff} \ \vartheta =\varrho ;$$
2. $${\mathcal {E}}_{s}(\vartheta ,\varrho )={\mathcal {E}}_{s}(\varrho ,\vartheta );$$
3. $${\mathcal {E}}_{s}(\vartheta ,\varrho )\le b[{\mathcal {E}}_{s}(\vartheta ,\omega )+{\mathcal {E}}_{s}(\omega ,\varrho )] +\gamma (\vartheta ,\varrho ){\mathcal {E}}_{s}(\vartheta ,\omega ) {\mathcal {E}}_{s}(\omega ,\varrho ).$$

An extended *b*-suprametric space is a pair $({\mathscr {M}},{\mathcal {E}}_{s})$ (shortly, ${\mathcal {E}}_{s}$-space), where ${\mathscr {M}}$ is a non-empty set and ${\mathcal {E}}_{s}$ is an extended *b*-suprametric.

### Example 1.2

Take ${\mathscr {M}}=\ell _{p}({\mathbb {R}})$ where as $p\in (0,1)$, and $\ell _{p}({\mathbb {R}})=\{\{\vartheta _{n}\}\subset {\mathbb {R}} \ \text {such that} \ \sum _{n=1}^{\infty }|\vartheta _{n}|^{p}<\infty \}$ and ${{\mathcal {E}}_{s}}_{\ell _{p}}:{\mathscr {M}}\times {\mathscr {M}}\rightarrow {\mathbb {R}}^{+}$ is provided that

$$\begin{aligned} {{\mathcal {E}}_{s}}_{\ell _{p}}(\{\vartheta _{n}\},\{\varrho _{n}\}) =\Big (\sum _{n=1}^{\infty }|\vartheta _{n}-\varrho _{n}|^{p}\Big )^{\frac{1}{p}} \ \text {for all} \ \{\vartheta _{n}\},\{\varrho _{n}\}\in {\mathscr {M}}. \end{aligned}$$

Then $({\mathscr {M}},{{\mathcal {E}}_{s}}_{\ell _{p}})$ is *b*-suprametric space with $b=2^{\frac{1}{p}}$.

Let ${\mathcal {E}}_{s}:{\mathscr {M}}\times {\mathscr {M}}\rightarrow {\mathbb {R}}^{+}$ and $\gamma :{\mathscr {M}}\times {\mathscr {M}}\rightarrow [1,\infty )$ defined by ${\mathcal {E}}_{s}(\vartheta ,\varrho )={{\mathcal {E}}_{s}}_{\ell _{p}} (\vartheta ,\varrho )[{{\mathcal {E}}_{s}}_{\ell _{p}}(\vartheta ,\varrho )+1]$ and $\gamma (\vartheta ,\varrho )=8^{\frac{1}{p}}\in [1,\infty ), \ 0<p<1$ with $b=4^{\frac{1}{p}}$. Then $({\mathscr {M}},{\mathcal {E}}_{s})$ is ${\mathcal {E}}_{s}$-space.

We give an overview of the generated topology in the next approach.

### Definition 1.3

Suppose we have ${\mathcal {E}}_{s}$-space $({\mathscr {M}},{\mathcal {E}}_{s})$. The set ${\mathscr {B}}(\vartheta _{0},r)=\{\vartheta \in {\mathscr {M}}/{\mathcal {E}}_{s}(\vartheta _{0},\vartheta )<r\}$ where $r>0$ and $\vartheta _{0}\in {\mathscr {M}}$ is called open ball. A subset ${\mathscr {N}}$ of ${\mathscr {M}}$ is called open whenever $\varrho \in {\mathscr {N}}$, there is a $r>0$ in such a way that ${\mathscr {B}}(\vartheta ,r)\subset {\mathscr {N}}$. $\tau$ will stand for the collection of every open subsets of ${\mathscr {M}}$.

### Proposition 1.4

Let $({\mathscr {M}},{\mathcal {E}}_{s})$ be a ${\mathcal {E}}_{s}$-space. Then each open ball is an open set.

### Proposition 1.5

Assume that there is ${\mathcal {E}}_{s}$-space $({\mathscr {M}},{\mathcal {E}}_{s})$ with $\gamma (\vartheta ,\varrho )=\beta \in [1,\infty )$, for all $\vartheta ,\varrho \in {\mathscr {M}}$. If $\varrho \in {\mathscr {B}}(\vartheta ,r)$, for $r>0$, then there exists $s>0$ such that ${\mathscr {B}}(\varrho ,s)\subseteq {\mathscr {B}}(\vartheta ,r)$.

### Proof

For all $\vartheta \in {\mathscr {M}}, \ r>0$ and ${\mathscr {B}}(\vartheta ,r)$ is non-empty. Now assume that $\vartheta \ne \varrho$, then we have ${\mathcal {E}}_{s}(\vartheta ,\varrho )\ne 0$. Choosing $s=\frac{r-b{\mathcal {E}}_{s}(\vartheta ,\varrho )}{b+\beta {\mathcal {E}}_{s}(\vartheta ,\varrho )}$ and let $\omega \in {\mathscr {B}}(\varrho ,s)$. Then owing to the property of the ${\mathcal {E}}_{s}$-space, we have

$$\begin{aligned} {\mathcal {E}}_{s}(\omega ,\vartheta )&\le b[{\mathcal {E}}_{s}(\omega ,\varrho )+{\mathcal {E}}_{s}(\varrho ,\vartheta )] +\gamma (\omega ,\vartheta ){\mathcal {E}}_{s} (\omega ,\varrho ){\mathcal {E}}_{s}(\varrho ,\vartheta )\\&<bs+b{\mathcal {E}}_{s}(\varrho ,\vartheta ) +\beta s{\mathcal {E}}_{s}(\varrho ,\vartheta )\\&=r, \end{aligned}$$

which yields, $\omega \in {\mathscr {B}}(\vartheta ,r)$. Thus, ${\mathscr {B}}(\varrho ,s)\subseteq {\mathscr {B}}(\vartheta ,r)$. Accordingly, an open subset of ${\mathscr {M}}$ is represented by each open ball. $\square$

### Proposition 1.6

$\tau$ defines a topology on $({\mathscr {M}},{\mathcal {E}}_{s})$ and the family of open balls form a base of the topology $\tau$.

### Proof

Let $\vartheta ,\varrho \in {\mathscr {M}}$ with $\vartheta \ne \varrho$ and $r={\mathcal {E}}_{s}(\vartheta ,\varrho )>0$. Denote ${\mathscr {U}}={\mathscr {B}}(\vartheta ,\frac{r}{2})$ and ${\mathscr {V}}={\mathscr {B}}(\varrho ,\frac{r(2-b)}{2b+\beta r})$ where $1\le b<2$.

Let us demonstrate that ${\mathscr {U}}\cap {\mathscr {V}}=\emptyset$, if none of the above applies, there is $\omega \in {\mathscr {U}}\cap {\mathscr {V}}$, as we have from hypothesis ${\mathcal {E}}_{s}(\vartheta ,\omega )<\frac{r}{2}$ and ${\mathcal {E}}_{s}(\varrho ,\omega )<\frac{r(2-b)}{2b+\beta r}$, we obtain,

$$\begin{aligned} r={\mathcal {E}}_{s}(\vartheta ,\varrho )&\le b[{\mathcal {E}}_{s}(\vartheta ,\omega ) +{\mathcal {E}}_{s}(\omega ,\varrho )]+\gamma (\vartheta ,\varrho ) {\mathcal {E}}_{s}(\vartheta ,\omega ){\mathcal {E}}_{s}(\omega ,\varrho ) \\&<b\Big [\frac{r}{2}+\frac{r(2-b)}{2b+\beta r}\Big ] +\beta \Big [\frac{r}{2}\frac{r(2-b)}{2b+\beta r}\Big ]\\&=r, \ \text {a contradiction}. \end{aligned}$$

Hence, ${\mathscr {U}}\cap {\mathscr {V}}=\emptyset$. As a result, ${\mathscr {M}}$ is Hausdorff. $\square$

### Definition 1.7

Let $({\mathscr {M}},{\mathcal {E}}_{s})$ be an ${\mathcal {E}}_{s}$-space. A sequence $\{\vartheta _{n}\}_{n\in {\mathbb {N}}}$ of elements of ${\mathscr {M}}$ Converges to $\vartheta \in {\mathscr {M}}$, if for every $\epsilon >0$ the ball ${\mathscr {B}}(\vartheta ,\epsilon )$ contained all that a finite number of terms of the sequence. In this case $\vartheta$ is a limit point of $\{\vartheta _{n}\}_{n\in {\mathbb {N}}}$ and we write $\lim _{n\rightarrow \infty }{\mathcal {E}}_{s}(\vartheta _{n},\vartheta )=0$.

### Proposition 1.8

Assume that there is ${\mathcal {E}}_{s}$-space $({\mathscr {M}},{\mathcal {E}}_{s})$. A sequence is unique if and only if $\{\vartheta _{n}\}_{n\in {\mathbb {N}}}\subset {\mathscr {M}}$ possesses a limit.

### Proof

One can easily deduce this result by using Hausdorffness. $\square$

### Definition 1.9

Assume that there is ${\mathcal {E}}_{s}$-space $({\mathscr {M}},{\mathcal {E}}_{s})$. A sequence $\{\vartheta _{n}\}_{n\in {\mathbb {N}}}\in {\mathscr {M}}$ is a Cauchy sequence if, for all $\epsilon >0$, there exists some $\kappa \in {\mathbb {N}}$ such that for all $n,m\ge \kappa , \ {\mathcal {E}}_{s}(\vartheta _{n},\vartheta _{m})<\epsilon$.

### Remark 1.10

If $\{\vartheta _{n}\}_{n\in {\mathbb {N}}}$ is a Cauchy sequence in $({\mathscr {M}},{\mathcal {E}}_{s})$ then there is a ${\mathfrak {q}}\in {\mathscr {M}}$ in such a way that $\lim _{n\rightarrow \infty }{\mathcal {E}}_{s}(\vartheta _{n},{\mathfrak {q}})=0$ and further every subsequence $\{\vartheta _{n(\kappa )}\}_{\kappa \in {\mathbb {N}}}$ converges to ${\mathfrak {q}}$.

### Definition 1.11

An ${\mathcal {E}}_{s}$-space $({\mathscr {M}},{\mathcal {E}}_{s})$ is called complete if every Cauchy sequence is convergent.

### Definition 1.12

Let $\Psi :{\mathscr {S}}\subset {\mathscr {M}}\rightarrow {\mathscr {M}}$ and there is $\vartheta _{0}\in {\mathscr {S}}$ in such a way that ${\mathscr {O}}(\vartheta _{0})=\{\vartheta _{0},\Psi \vartheta _{0}, \Psi ^{2}\vartheta _{0},....\}\subset {\mathscr {S}}$. An orbit of $\vartheta _{0}\in {\mathscr {S}}$ is denoted by the set ${\mathscr {O}}(\vartheta _{0})$. A function ${\mathscr {G}}$ from ${\mathscr {S}}$ into the collection of real numbers is predominantly called $\Psi$-orbitally lower semicontinuous at $t\in {\mathscr {S}}$ whenever $\{\vartheta _{n}\}\subset {\mathscr {O}}(\vartheta _{0})$ and $\vartheta _{n}\rightarrow t$ $\Rightarrow$ ${\mathscr {G}}(t)\le \liminf _{n\rightarrow \infty }{\mathscr {G}}(\vartheta _{n})$.

We can observe that J. Matkowski^24^ introduced the concept of comparison functions initially. Subsequently, various modifications and extensions of these comparison functions are provided to complete their outcomes (see,^25–27^).

Now, inspired by the above literature, we introduce the below definition.

### Definition 1.13

Assume that there is ${\mathcal {E}}_{s}$-space $({\mathscr {M}},{\mathcal {E}}_{s})$. A function $\psi :{\mathbb {R}}^{+}\rightarrow {\mathbb {R}}^{+}$ is an extended *b*-supra-comparison function (shortly, $E_{sC}$-function) if it is increasing and there exist a mapping $\Psi :{\mathscr {S}}\subset {\mathscr {M}}\rightarrow {\mathscr {M}}$ such that for some $\vartheta _{0}\in {\mathscr {S}}$ and ${\mathscr {O}}(\vartheta _{0})\subset {\mathscr {S}}$,

$$\begin{aligned} \sum _{n=0}^{\infty }\psi ^{n}(t)\prod _{i=1}^{n} \left[b+\gamma (\vartheta _{i},\vartheta _{m})\psi ^{n}(t)\right], \end{aligned}$$

converges. Here $\vartheta _{n}=\Psi ^{n}\vartheta _{0}, \forall n=1,2,3...$ Then, $\psi$ is an $E_{sC}$-function for $\Psi$ at $\vartheta _{0}$.

## Results on ${\mathcal {E}}_{s}$-space

### Theorem 2.1

Let $({\mathscr {M}},{\mathcal {E}}_{s})$ be a complete ${\mathcal {E}}_{s}$-space and $\Psi :{\mathscr {M}}\rightarrow {\mathscr {M}}$ be a mapping. Take $\eta \in [0,1)$ in such a way that

2.1 $$\begin{aligned} {\mathcal {E}}_{s}(\Psi \vartheta ,\Psi \varrho )\le \frac{\eta }{b}{\mathcal {E}}_{s}(\vartheta ,\varrho ), \end{aligned}$$

for all $\vartheta ,\varrho \in {\mathscr {M}}$. Then $\Psi$ has a unique fixed point, and for every $\vartheta _{0}\in {\mathscr {M}}$ the iterative sequence defined by $\vartheta _{n}=\Psi \vartheta _{n-1}, \ \forall n\in {\mathbb {N}}$ converges to this fixed point.

### Proof

Let $({\mathscr {M}},{\mathcal {E}}_{s})$ be a complete ${\mathcal {E}}_{s}$-space. Define the sequence $\{\vartheta _{n}\}$ by $\vartheta _{n}=\Psi \vartheta _{n-1}, \ \forall n\in {\mathbb {N}}$ for some arbitrary $\vartheta _{0}\in {\mathscr {M}}$. Now from (2.1), we deduce that

$$\begin{aligned} {\mathcal {E}}_{s}(\vartheta _{n},\vartheta _{n+1})&={\mathcal {E}}_{s}(\Psi \vartheta _{n-1},\Psi \vartheta _{n})\\&\le \frac{\eta }{b}{\mathcal {E}}_{s}(\vartheta _{n-1},\vartheta _{n})\\&<{\mathcal {E}}_{s}(\vartheta _{n-1},\vartheta _{n}).\\ \end{aligned}$$

Thus, regardless of the given integer $\kappa$, the sequence $\{{\mathcal {E}}_{s}(\vartheta _{n},\vartheta _{n+1})\}$ is non-increasing and meets the following:

2.2 $$\begin{aligned} {\mathcal {E}}_{s}(\vartheta _{n},\vartheta _{n+1})\le \Big (\frac{\eta }{b}\Big )^{n-\kappa }{\mathcal {E}}_{s} (\vartheta _{\kappa },\vartheta _{\kappa +1}), \ \forall n>\kappa . \end{aligned}$$

Therefore, $\lim _{n\rightarrow \infty }{\mathcal {E}}_{s}(\vartheta _{n},\vartheta _{n+1})=0,$ which yields that for $\epsilon >0, \ \kappa \in {\mathbb {N}}$ such that for all $n\ge \kappa$, we have

2.3 $$\begin{aligned} {\mathcal {E}}_{s}(\vartheta _{n},\vartheta _{n+1})<\epsilon . \end{aligned}$$

We shall now demonstrate the Cauchy nature of the series $\{\vartheta _{n}\}$. BY utilizing (2.2), (2.3) and triangular inequality, we have

2.4 $$\begin{aligned} {\mathcal {E}}_{s}(\vartheta _{\mathbbm {p}},\vartheta _{\mathbbm {q}})&\le b\big [{\mathcal {E}}_{s}(\vartheta _{\mathbbm {p}},\vartheta _{\mathbbm {p}+1}) +{\mathcal {E}}_{s}(\vartheta _{\mathbbm {p}+1},\vartheta _{\mathbbm {q}})\big ] +\gamma (\vartheta _{\mathbbm {p}},\vartheta _{\mathbbm {q}}){\mathcal {E}}_{s} (\vartheta _{\mathbbm {p}},\vartheta _{\mathbbm {p}+1}){\mathcal {E}}_{s} (\vartheta _{\mathbbm {p}+1},\vartheta _{\mathbbm {q}})\nonumber \\&\le b\Big [\Big (\frac{\eta }{b}\Big )^{\mathbbm {p}-\kappa }{\mathcal {E}}_{s}( \vartheta _{\mathbbm {p}},\vartheta _{\mathbbm {p}+1})+{\mathcal {E}}_{s}(\vartheta _ {\mathbbm {p}+1},\vartheta _{\mathbbm {q}})\Big ]+\gamma (\vartheta _{\mathbbm {p}}, \vartheta _{\mathbbm {q}})\Big (\frac{\eta }{b}\Big )^{\mathbbm {p}-\kappa } {\mathcal {E}}_{s}(\vartheta _{\mathbbm {p}},\vartheta _{\mathbbm {p}+1}) {\mathcal {E}}_{s}(\vartheta _{\mathbbm {p}+1},\vartheta _{\mathbbm {q}})\nonumber \\&\le b\Big [\Big (\frac{\eta }{b}\Big )^{\mathbbm {p}-\kappa }\epsilon +{\mathcal {E}}_{s}(\vartheta _{\mathbbm {p}+1},\vartheta _{\mathbbm {q}})\Big ] +\gamma (\vartheta _{\mathbbm {p}},\vartheta _{\mathbbm {q}})\Big (\frac{\eta }{b}\Big ) ^{\mathbbm {p}-\kappa }\epsilon {\mathcal {E}}_{s}(\vartheta _{\mathbbm {p}+1}, \vartheta _{\mathbbm {q}})\nonumber \\&\le b\Big (\frac{\eta }{b}\Big )^{\mathbbm {p}-\kappa }\epsilon +\Bigg [b+\gamma (\vartheta _{\mathbbm {p}},\vartheta _{\mathbbm {q}}) \Big (\frac{\eta }{b}\Big )^{\mathbbm {p}-\kappa }\epsilon \Bigg ] {\mathcal {E}}_{s}(\vartheta _{\mathbbm {p}+1},\vartheta _{\mathbbm {q}}), \end{aligned}$$

where,

2.5 $$\begin{aligned} {\mathcal {E}}_{s}(\vartheta _{\mathbbm {p}+1},\vartheta _{\mathbbm {q}})&\le b\big [{\mathcal {E}}_{s}(\vartheta _{\mathbbm {p}+1},\vartheta _{\mathbbm {p}+2}) +{\mathcal {E}}_{s}(\vartheta _{\mathbbm {p}+2},\vartheta _{\mathbbm {q}})\big ] +\gamma (\vartheta _{\mathbbm {p}+1},\vartheta _{\mathbbm {q}}) {\mathcal {E}}_{s}(\vartheta _{\mathbbm {p}+1},\vartheta _{\mathbbm {p}+2}) {\mathcal {E}}_{s}(\vartheta _{\mathbbm {p}+2},\vartheta _{\mathbbm {q}})\nonumber \\&\le b\Big [\Big (\frac{\eta }{b}\Big )^{\mathbbm {p}-\kappa +1}\epsilon +{\mathcal {E}}_{s}(\vartheta _{\mathbbm {p}+2},\vartheta _{\mathbbm {q}})\Big ] +\gamma (\vartheta _{\mathbbm {p}+1},\vartheta _{\mathbbm {q}})\Big (\frac{\eta }{b}\Big ) ^{\mathbbm {p}-\kappa +1}\epsilon {\mathcal {E}}_{s}(\vartheta _{\mathbbm {p}+2} ,\vartheta _{\mathbbm {q}})\nonumber \\&\le b\Big (\frac{\eta }{b}\Big )^{\mathbbm {p}-\kappa +1}\epsilon +\Bigg [b+\gamma (\vartheta _{\mathbbm {p}+1},\vartheta _{\mathbbm {q}}) \Big (\frac{\eta }{b}\Big )^{\mathbbm {p}-\kappa +1}\epsilon \Bigg ] {\mathcal {E}}_{s}(\vartheta _{\mathbbm {p}+2},\vartheta _{\mathbbm {q}}). \end{aligned}$$

From the above two inequalities (2.4) and (2.5), we get,

$$\begin{aligned} {\mathcal {E}}_{s}(\vartheta _{\mathbbm {p}},\vartheta _{\mathbbm {q}})&\le b\Big (\frac{\eta }{b}\Big )^{\mathbbm {p}-\kappa }\epsilon +b\Big (\frac{\eta }{b}\Big )^{\mathbbm {p}-\kappa +1}\epsilon \Big [b+\gamma (\vartheta _{\mathbbm {p}},\vartheta _{\mathbbm {q}}) \Big (\frac{\eta }{b}\Big )^{\mathbbm {p}-\kappa }\epsilon \Big ]\\&\ \ \ \ \ \ \ +\Big [b+\gamma (\vartheta _{\mathbbm {p}}, \vartheta _{\mathbbm {q}})\Big (\frac{\eta }{b}\Big )^{\mathbbm {p} -\kappa }\epsilon \Big ]\Big [b+\gamma (\vartheta _{\mathbbm {p}+1}, \vartheta _{\mathbbm {q}})\Big (\frac{\eta }{b}\Big )^{\mathbbm {p} -\kappa +1}\epsilon \Big ]{\mathcal {E}}_{s}(\vartheta _{\mathbbm {p}+2}, \vartheta _{\mathbbm {q}}) \end{aligned}$$

Employing (2.3) in each of the terms in the sum, we can keep proceeding until we get

$$\begin{aligned} {\mathcal {E}}_{s}(\vartheta _{\mathbbm {p}},\vartheta _{\mathbbm {q}})&\le b\Big (\frac{\eta }{b}\Big )^{\mathbbm {p}-\kappa }\epsilon +b\Big (\frac{\eta }{b}\Big )^{\mathbbm {p}-\kappa +1}\epsilon \Big [b +\gamma (\vartheta _{\mathbbm {p}},\vartheta _{\mathbbm {q}})\Big (\frac{\eta }{b}\Big ) ^{\mathbbm {p}-\kappa }\epsilon \Big ]\\&\ \ \ \ \ \ \ +b\Big (\frac{\eta }{b}\Big )^{\mathbbm {p}-\kappa +2} \epsilon \Big [b+\gamma (\vartheta _{\mathbbm {p}},\vartheta _{\mathbbm {q}}) \Big (\frac{\eta }{b}\Big )^{\mathbbm {p}-\kappa }\epsilon \Big ] \Big [b+\gamma (\vartheta _{\mathbbm {p}+1},\vartheta _{\mathbbm {q}}) \Big (\frac{\eta }{b}\Big )^{\mathbbm {p}-\kappa +1}\epsilon \Big ]+\cdots \\&\le b\Big (\frac{\eta }{b}\Big )^{\mathbbm {p}-\kappa }\epsilon \sum _{i=0}^{\mathbbm {q}-\mathbbm {p}-1} \Big (\frac{\eta }{b}\Big )^{i} \prod _{j=0}^{i-1}\Big [b+\epsilon \gamma (\vartheta _{\mathbbm {p}+j}, \vartheta _{\mathbbm {q}})\Big (\frac{\eta }{b}\Big )^{\mathbbm {p}-\kappa +j}\Big ] \end{aligned}$$

Since $\frac{\eta }{b}\in [0,1)$, it follows that

$$\begin{aligned} {\mathcal {E}}_{s}(\vartheta _{\mathbbm {p}},\vartheta _{\mathbbm {q}})\le b\Big (\frac{\eta }{b}\Big )^{\mathbbm {p}-\kappa } \epsilon \sum _{i=0}^{\mathbbm {q}-\mathbbm {p}-1} \Big (\frac{\eta }{b}\Big )^{i}\prod _{j=0}^{i-1}\Big [b+\epsilon \gamma (\vartheta _{\mathbbm {p}+j},\vartheta _{\mathbbm {q}}) \Big (\frac{\eta }{b}\Big )^{j}\Big ]. \end{aligned}$$

Now, one can easily verify that the series $\sum _{i=0}^{\infty }U_{i}$ converges by ratio test, where,

$$\begin{aligned} U_{i}=\Big (\frac{\eta }{b}\Big )^{i}\prod _{j=0}^{i-1}\Big [b+\epsilon \gamma (\vartheta _{\mathbbm {p}+j},\vartheta _{\mathbbm {q}}) \Big (\frac{\eta }{b}\Big )^{j}\Big ]. \end{aligned}$$

Hence we deduce that ${\mathcal {E}}_{s}(\vartheta _{\mathbbm {p}},\vartheta _{\mathbbm {q}})\rightarrow 0$ as $\mathbbm {p},\mathbbm {q}$ tends to infinity, thus, $\{\vartheta _{n}\}$ is Cauchy. $\{\vartheta _{n}\}$ converges to some ${\mathfrak {q}}\in {\mathscr {M}}$ as per the completeness of ${\mathscr {M}}$, it follows that every subsequence $\{\vartheta _{n(\kappa )}\}_{\kappa \in {\mathbb {N}}}$ converges to ${\mathfrak {q}}$. Let us now assert that ${\mathfrak {q}}$ is a fixed point of $\Psi$. BY using (2.1) and continuity of $\Psi$, we get,

$$\begin{aligned} {\mathcal {E}}_{s}(\Psi \vartheta _{n(\kappa )},\Psi {\mathfrak {q}})\le \eta {\mathcal {E}}_{s}(\vartheta _{n(\kappa )},{\mathfrak {q}}). \end{aligned}$$

Therefore, we conclude that ${\mathfrak {q}}=\Psi {\mathfrak {q}}$ as $\kappa$ attains infinity.

Hence ${\mathfrak {q}}$ is a fixed point of $\Psi$.

In order to prove uniqueness, assume that $\vartheta _{1}$ and $\vartheta _{2}$ are two fixed points which are distinct. Which yields that ${\mathcal {E}}_{s}(\vartheta _{1},\vartheta _{2})\ne 0$. From (2.1), we obtain,

$$\begin{aligned} {\mathcal {E}}_{s}(\vartheta _{1},\vartheta _{2})={\mathcal {E}}_{s} (\Psi \vartheta _{1},\Psi \vartheta _{2})\le \frac{\eta }{b}{\mathcal {E}}_{s}(\vartheta _{1},\vartheta _{2}) <{\mathcal {E}}_{s}(\vartheta _{1},\vartheta _{2}), \end{aligned}$$

which is absurd, and therefore $\vartheta _{1}=\vartheta _{2}$. $\square$

### Example 2.2

Let ${\mathscr {M}}=[0,\pi ]$. Define a mapping ${\mathcal {E}}_{s}:{\mathscr {M}}\times {\mathscr {M}}\rightarrow [0,\infty )$ by ${\mathcal {E}}_{s}(\vartheta ,\varrho )=|\vartheta -\varrho |$ for all $\vartheta ,\varrho \in {\mathscr {M}}$ and $\gamma :{\mathscr {M}}\times {\mathscr {M}}\rightarrow [1,\infty )$ by $\gamma (\vartheta ,\varrho )=e^{\vartheta +\varrho }+1$ for all $\vartheta ,\varrho \in {\mathscr {M}}$. Clearly $({\mathscr {M}},{\mathcal {E}}_{s})$ is a complete ${\mathcal {E}}_{s}$-space.

Define a mapping $\Psi :{\mathscr {M}}\rightarrow {\mathscr {M}}$ by

$$\begin{aligned} \Psi \vartheta =\frac{1}{2}\sin \vartheta +\frac{1}{1+\sin \vartheta }, \ \text {for all} \ \vartheta \in {\mathscr {M}}. \end{aligned}$$

Now lets prove that $\Psi$ satisfies (2.1) of Theorem.2.1.

Consider

2.6 $$\begin{aligned} |\Psi (\vartheta )-\Psi (\varrho )|&=\Big |\frac{1}{2}(\sin \vartheta -\sin \varrho ) +\frac{1}{1+\sin \vartheta }-\frac{1}{1+\sin \varrho }\Big | \nonumber \\&=\Big |\frac{1}{2}(\sin \vartheta -\sin \varrho ) +\frac{\sin \varrho -\sin \vartheta }{(1+\sin \vartheta )(1+\sin \varrho )}\Big | \nonumber \\&\le \frac{1}{2}|\vartheta -\varrho |+\frac{|\vartheta -\varrho |}{(1+\sin \vartheta )(1+\sin \varrho )} \end{aligned}$$

By using the boundedness of the function $\sin \vartheta$ in the interval $[0,\pi ]$, there exists a constant $\Lambda >0$ such that $\frac{1}{(1+\sin \vartheta )(1+\sin \varrho )}\le \Lambda$. Thus from the above inequality (2.6), we obtain

$$\begin{aligned} |\Psi (\vartheta )-\Psi (\varrho )|&\le \frac{1}{2}|\vartheta -\varrho |+\Lambda |\vartheta -\varrho |\\&\le \bigg [\frac{1}{2}+\Lambda \bigg ]|-\varrho |\\&=\frac{\eta }{b}|\vartheta -\varrho |, \ \text {where} \ \frac{\eta }{b} =\frac{1}{2}+\Lambda <1 \ \text {for sufficiently small} \ \Lambda . \end{aligned}$$

**Figure 1:**
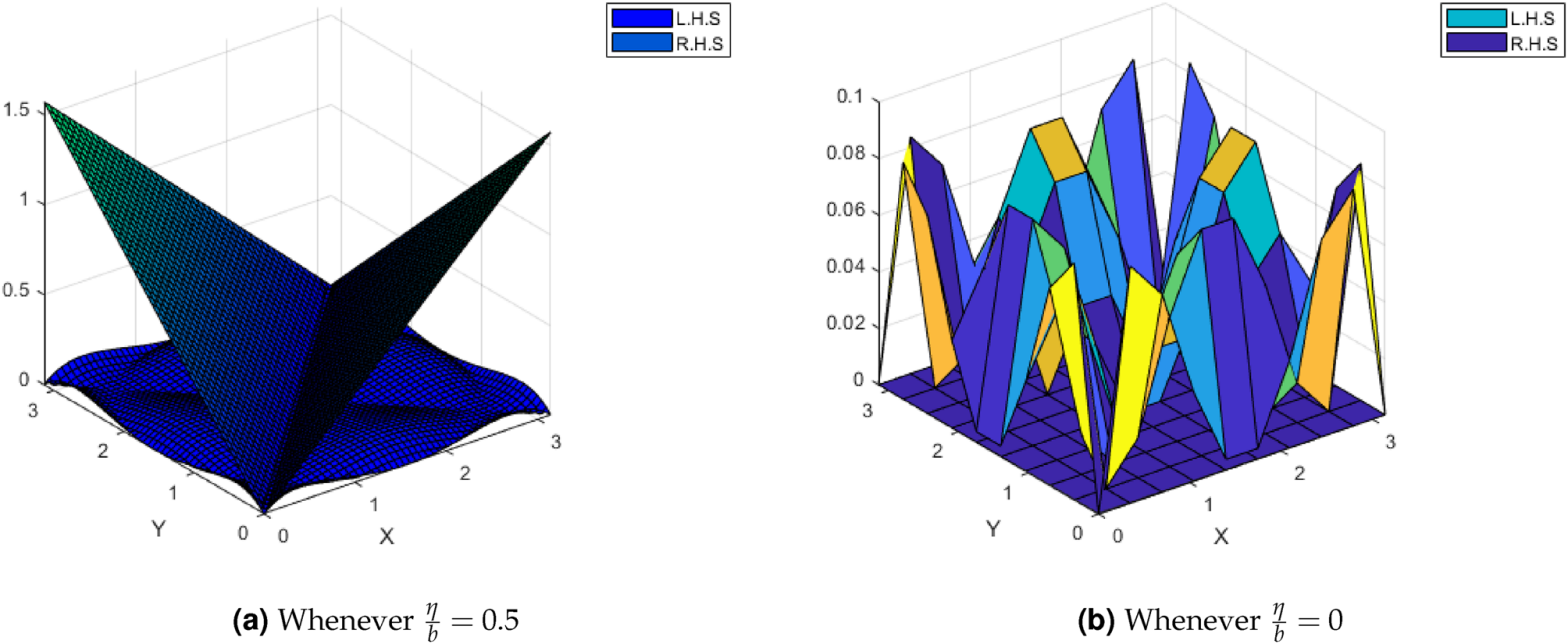
Comparison of L.H.S and R.H.S of Inequality (2.1) of Theorem.2.1.

Hence all the conditions of Theorem.2.1 satisfied and 0.9592 is the unique fixed point of $\Psi$, which is obtained with the help of MATLAB with $\frac{\eta }{b}\in [0,1)$. Moreover, the comparisons of L.H.S and R.H.S of the contraction (2.1) of Theorem.2.1 using MATLAB for this example as shown in the Fig. 1.

**Figure 2:**
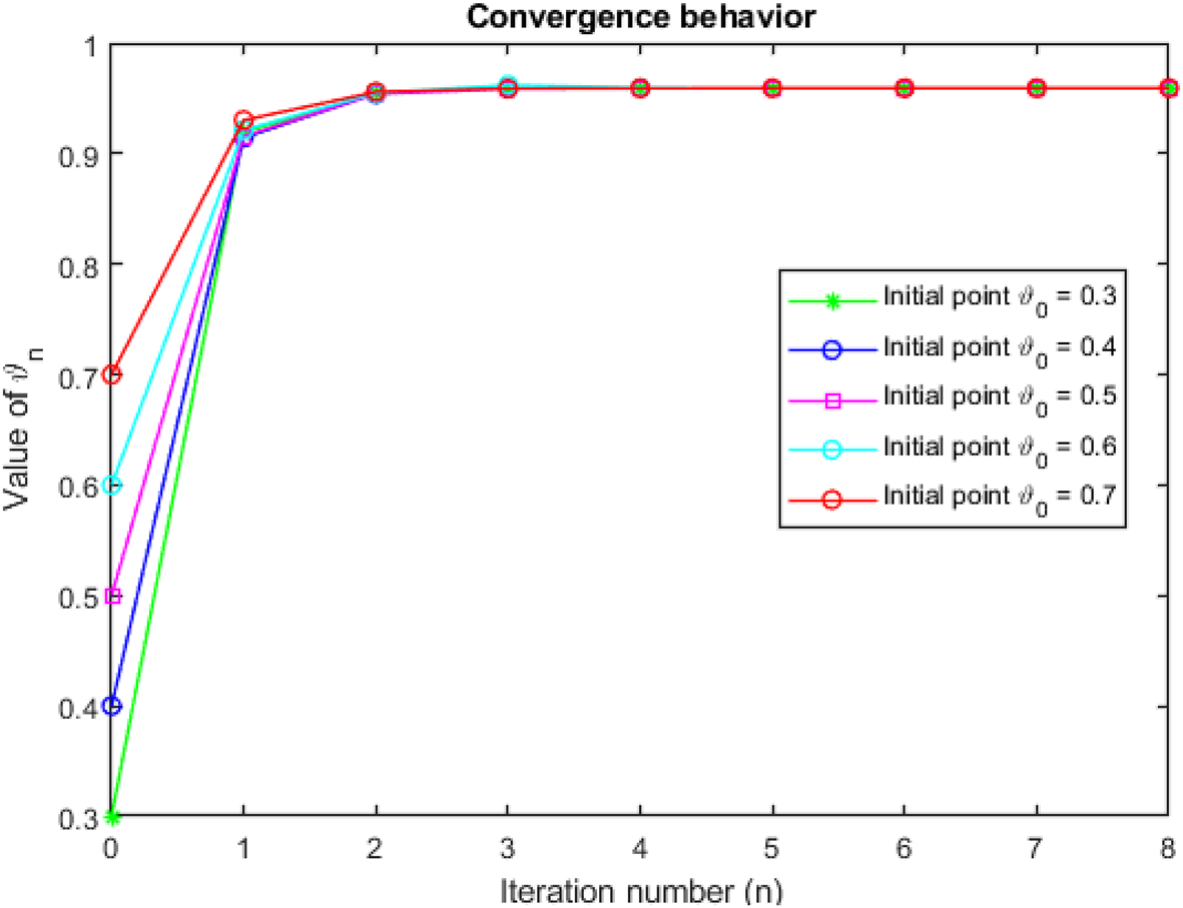
Convergence behavior for Example 2.2.

Now, we perform a few numerical simulations to estimate $\Psi$’s fixed point in Table 1. Additionally, Fig. 2 illustrates how the aforementioned iterations converge.

**Table 1:** Picard iterations.

$\vartheta _{0}$	$\vartheta _{0}=0.3$	$\vartheta _{0}=0.4$	$\vartheta _{0}=0.5$	$\vartheta _{0}=0.6$	$\vartheta _{0}=0.7$
$\vartheta _{1}$	0.919651	0.914435	0.915651	0.921445	0.930301
$\vartheta _{2}$	0.954677	0.954077	0.954217	0.954883	0.955901
$\vartheta _{3}$	0.958674	0.958618	0.958633	0.962096	0.958825
$\vartheta _{4}$	0.95914	0.959134	0.959135	0.959528	0.959157
$\vartheta _{5}$	0.959193	0.959192	0.959192	0.959237	0.959195
$\vartheta _{6}$	0.959199	0.959199	0.959199	0.959204	0.959199
$\vartheta _{7}$	0.9592	0.9592	0.9592	0.9592	0.9592
$\vartheta _{8}$	0.9592	0.9592	0.9592	0.9592	0.9592

### Example 2.3

Let ${\mathscr {M}}=[0,\pi ]$. Define a mapping ${\mathcal {E}}_{s}:{\mathscr {M}}\times {\mathscr {M}}\rightarrow [0,\infty )$ by ${\mathcal {E}}_{s}(\vartheta ,\varrho )=\bigg |\frac{\sin (\vartheta -\varrho )}{1+\sin \vartheta }\bigg |$ for all $\vartheta ,\varrho \in {\mathscr {M}}$ and $\gamma :{\mathscr {M}}\times {\mathscr {M}}\rightarrow [1,\infty )$ by $\gamma (\vartheta ,\varrho )=e^{\vartheta +\varrho }$ for all $\vartheta ,\varrho \in {\mathscr {M}}$. Clearly $({\mathscr {M}},{\mathcal {E}}_{s})$ is a complete ${\mathcal {E}}_{s}$-space.

Define a mapping $\Psi :{\mathscr {M}}\rightarrow {\mathscr {M}}$ by

$$\begin{aligned} \Psi \vartheta =\frac{1}{2}\sin \vartheta +\frac{1}{1+\sin \vartheta }, \ \text {for all} \ \vartheta \in {\mathscr {M}}. \end{aligned}$$

**Figure 3:**
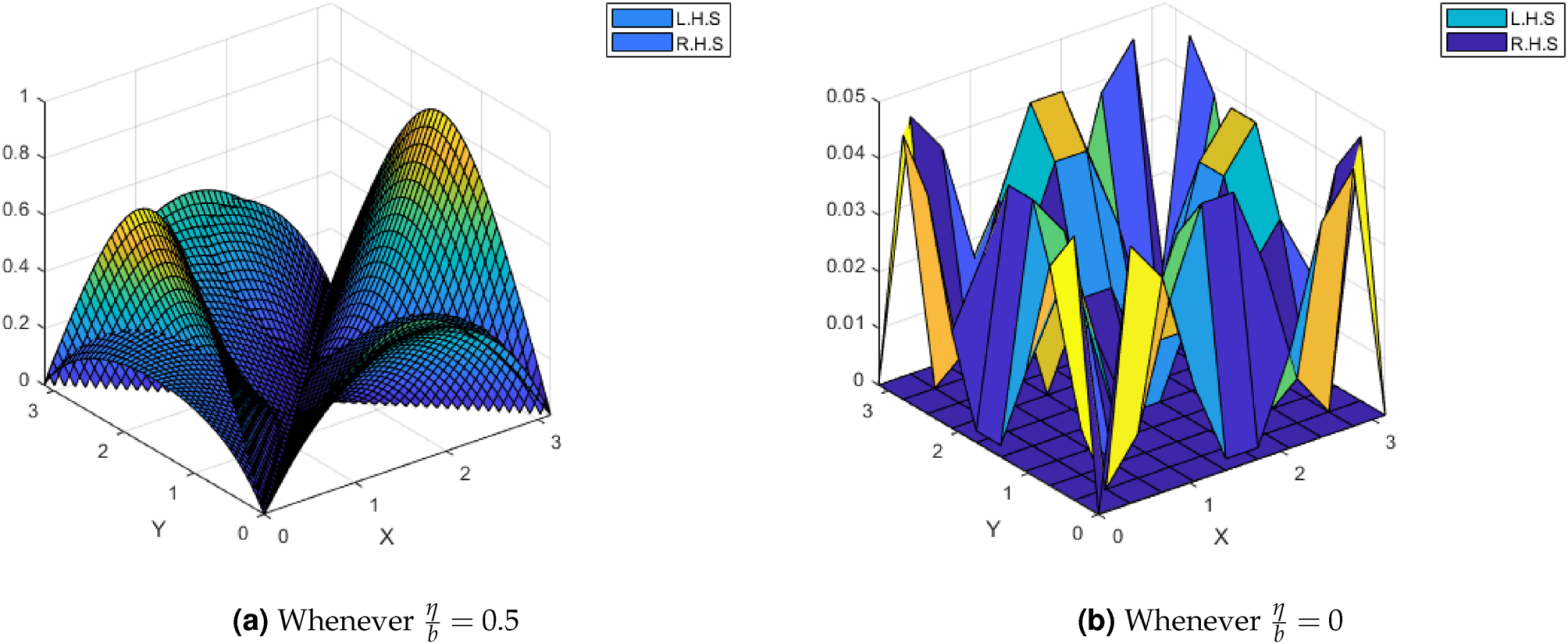
Comparison of L.H.S and R.H.S of Inequality (2.1) of Theorem.2.1.

By following the same pattern as above, we can easily deduce that $\Psi (\vartheta )$ satisfies Eq. (2.1) of Theorem 2.1. By using the numerical method in MATLAB, we obtain the unique fixed point of $\Psi$ which is 0.9592 lies in the interval $[0,\pi ]$. Thus, this example illustrates our Theorem 2.1. Moreover, the comparisons of L.H.S and R.H.S of the contraction (2.1) of Theorem 2.1 using MATLAB for this example are shown in the Fig. 3.

### Theorem 2.4

Let $({\mathscr {M}},{\mathcal {E}}_{s})$ be a complete ${\mathcal {E}}_{s}$-space such that ${\mathcal {E}}_{s}$ is continuous. Consider the mapping $\Psi :{\mathscr {S}}\subset {\mathscr {M}}\rightarrow {\mathscr {M}}$ such that ${\mathscr {O}}(\vartheta _{0})\subset {\mathscr {S}}$. Assume that,

2.7 $$\begin{aligned} {\mathcal {E}}_{s}(\Psi \vartheta , \Psi ^{2}\vartheta )\le \psi ({\mathcal {E}}_{s}(\vartheta ,\Psi \vartheta )), \end{aligned}$$

for each $\vartheta \in {\mathscr {O}}(\vartheta _{0})$, where $\psi$ is an $E_{sC}$-function for $\Psi$ at $\vartheta _{0}$. Then $\Psi ^{n}\vartheta _{0}\rightarrow \delta \in {\mathscr {M}}$. Furthermore, $\delta$ is a fixed point of $\Psi$ iff ${\mathscr {G}}(\vartheta )={\mathcal {E}}_{s}(\vartheta ,\Psi \vartheta )$ is $\Psi$-orbitally lower semicontinuous at $\delta$.

### Proof

Assume that $({\mathscr {M}},{\mathcal {E}}_{s})$ is a complete ${\mathcal {E}}_{s}$-space. Define the iterative sequence $\{\vartheta _{n}\}$ by $\vartheta _{0},\psi \vartheta _{0}=\vartheta _{1},\vartheta _{2} =\Psi \vartheta _{1}=\Psi (\Psi \vartheta _{0})=\Psi ^{2}(\vartheta _{0}) ....\vartheta _{n}=\Psi ^{n}(\vartheta _{0})...$ $\forall n\in {\mathbb {N}}$ and $\vartheta _{0}\in {\mathscr {M}}$.

In the remaining portions of the convincing proof we attempt to streamline the idea ${{\mathcal {E}}_{s}}_{m,n}={\mathcal {E}}_{s}(\vartheta _{m},\vartheta _{n})$ and $\gamma _{m,n}=\gamma (\vartheta _{m},\vartheta _{n})$ for all $m,n\in {\mathbb {N}}$.

By successively applying inequality (2.7), we obtain,

2.8 $$\begin{aligned} {{\mathcal {E}}_{s}}_{n,n+1}\le \psi ^{n}({{\mathcal {E}}_{s}}_{0,1}). \end{aligned}$$

By triangular inequality and (2.8), for $\mathbbm {p}>\mathbbm {q}$ we have

2.9 $$\begin{aligned} {{\mathcal {E}}_{s}}_{\mathbbm {p},\mathbbm {q}}&\le b[{{\mathcal {E}}_{s}}_{\mathbbm {p},\mathbbm {p}+1} +{{\mathcal {E}}_{s}}_{\mathbbm {p}+1,\mathbbm {q}}]+\gamma _{\mathbbm {p}, \mathbbm {q}}{{\mathcal {E}}_{s}}_{\mathbbm {p},\mathbbm {p}+1}{{\mathcal {E}}_{s}}_ {\mathbbm {p}+1,\mathbbm {q}}\nonumber \\&\le b\psi ^{\mathbbm {p}}({{\mathcal {E}}_{s}}_{0,1})+b{{\mathcal {E}}_{s}}_ {\mathbbm {p}+1,\mathbbm {q}}+\gamma _{\mathbbm {p},\mathbbm {q}}\psi ^ {\mathbbm {p}}({{\mathcal {E}}_{s}}_{0,1}){{\mathcal {E}}_{s}}_{\mathbbm {p}+1, \mathbbm {q}}\nonumber \\&\le b\psi ^{\mathbbm {p}}({{\mathcal {E}}_{s}}_{0,1})+[b+\gamma _{\mathbbm {p}, \mathbbm {q}}\psi ^{\mathbbm {p}}({{\mathcal {E}}_{s}}_{0,1})]{{\mathcal {E}}_{s}}_ {\mathbbm {p}+1,\mathbbm {q}} \end{aligned}$$

where

2.10 $$\begin{aligned} {{\mathcal {E}}_{s}}_{\mathbbm {p}+1,\mathbbm {q}}&\le b[{{\mathcal {E}}_{s}}_ {\mathbbm {p+1},\mathbbm {p}+2}+{{\mathcal {E}}_{s}}_{\mathbbm {p}+2, \mathbbm {q}}]+\gamma _{\mathbbm {p}+1,\mathbbm {q}}{{\mathcal {E}}_{s}}_ {\mathbbm {p}+1,\mathbbm {p}+2}{{\mathcal {E}}_{s}}_{\mathbbm {p}+2,\mathbbm {q}}\nonumber \\&\le b\psi ^{\mathbbm {p}+1}({{\mathcal {E}}_{s}}_{0,1})+[b+\gamma _{\mathbbm {p} +1,\mathbbm {q}}\psi ^{\mathbbm {p}+1}({{\mathcal {E}}_{s}}_{0,1})]{{\mathcal {E}}_{s}}_ {\mathbbm {p}+2,\mathbbm {q}} \end{aligned}$$

By combining the previous two inequalities (2.9) and (2.10), we obtain

2.11 $$\begin{aligned} {{\mathcal {E}}_{s}}_{\mathbbm {p},\mathbbm {q}}&\le b\psi ^{\mathbbm {p}}({{\mathcal {E}}_{s}}_{0,1})+b\psi ^{\mathbbm {p}+1} ({{\mathcal {E}}_{s}}_{0,1})+[b+\gamma _{\mathbbm {p},\mathbbm {q}} \psi ^{\mathbbm {p}}({{\mathcal {E}}_{s}}_{0,1})]\nonumber \\&\ \ \ \ \ \ +[b+\gamma _{\mathbbm {p},\mathbbm {q}}\psi ^{\mathbbm {p}} ({{\mathcal {E}}_{s}}_{0,1})][b+\gamma _{\mathbbm {p}+1,\mathbbm {q}}\psi ^{\mathbbm {p}+1} ({{\mathcal {E}}_{s}}_{0,1})]{{\mathcal {E}}_{s}}_{\mathbbm {p}+2,\mathbbm {q}} \end{aligned}$$

By continuing this process until we obtain

$$\begin{aligned} {{\mathcal {E}}_{s}}_{\mathbbm {p},\mathbbm {q}}&\le b\psi ^{\mathbbm {p}}({{\mathcal {E}}_{s}}_{0,1})+b\psi ^{\mathbbm {p}+1} ({{\mathcal {E}}_{s}}_{0,1})+[b+\gamma _{\mathbbm {p},\mathbbm {q}} \psi ^{\mathbbm {p}}({{\mathcal {E}}_{s}}_{0,1})]\\&\ \ \ \ \ \ +b\psi ^{\mathbbm {p}+2}({{\mathcal {E}}_{s}}_{0,1}) [b+\gamma _{\mathbbm {p},\mathbbm {q}}\psi ^{\mathbbm {p}}({{\mathcal {E}}_{s}}_{0,1})] [b+\gamma _{\mathbbm {p}+1,\mathbbm {q}}\psi ^{\mathbbm {p}+1} ({{\mathcal {E}}_{s}}_{0,1})]+\cdots \nonumber \\&\ \ \ \ \ \ \cdots +b\psi ^{\mathbbm {q}-1}({{\mathcal {E}}_{s}}_{0,1}) [b+\gamma _{\mathbbm {p},\mathbbm {q}}\psi ^{\mathbbm {p}}({{\mathcal {E}}_{s}}_{0,1})] [b+\gamma _{\mathbbm {p}+1,\mathbbm {q}}\psi ^{\mathbbm {p}+1}({{\mathcal {E}}_{s}}_{0,1})] \cdots [b+\gamma _{\mathbbm {q}-2,\mathbbm {q}}\psi ^{\mathbbm {q}-2}({{\mathcal {E}}_{s}}_{0,1})]\\&\le b\sum _{i=0}^{\mathbbm {q}-\mathbbm {p}-1}\psi ^{\mathbbm {p}+i}({{\mathcal {E}}_{s}}_{0,1}) \prod _{j=0}^{i-1}[b+\gamma _{\mathbbm {p}+j,\mathbbm {q}}\psi ^{\mathbbm {p}+j}({{\mathcal {E}}_{s}}_{0,1})]\\&\le b\sum _{i=0}^{\infty }\psi ^{\mathbbm {p}+i}({{\mathcal {E}}_{s}}_{0,1})\prod _{j=0}^{i-1} [b+\gamma _{\mathbbm {p}+j,\mathbbm {q}}\psi ^{\mathbbm {p}+j}({{\mathcal {E}}_{s}}_{0,1})]. \end{aligned}$$

The series

$$\begin{aligned} \sum _{i=0}^{\infty }\psi ^{\mathbbm {p}+i}({{\mathcal {E}}_{s}}_{0,1})\prod _{j=0}^{i-1} \left[b+\gamma _{\mathbbm {p}+j,\mathbbm {q}}\psi ^{\mathbbm {p}+j}({{\mathcal {E}}_{s}}_{0,1})\right], \end{aligned}$$

converges for each $\mathbbm {p},\mathbbm {q}\in {\mathbb {N}}$. Thus, we conclude that $\{\vartheta _{n}\}$ is Cauchy since ${{\mathcal {E}}_{s}}_{\mathbbm {p},\mathbbm {q}}\rightarrow 0$ as $\mathbbm {p},\mathbbm {q}\rightarrow \infty$. Since ${\mathscr {M}}$ is complete then $\vartheta _{n}=\Psi ^{n}\vartheta _{0}\rightarrow \delta \in {\mathscr {M}}$. Given the assumption the fact that ${\mathscr {G}}$ is semicontinuous at $\delta \in {\mathscr {M}}$, implies that,

$$\begin{aligned} {\mathcal {E}}_{s}(\delta ,\Psi \delta )&\le \liminf _{n\rightarrow \infty }{\mathcal {E}}_{s} (\Psi ^{n}\vartheta _{0},\Psi ^{n+1}\vartheta _{0})\\&\le \liminf _{n\rightarrow \infty } \psi ^{n}({{\mathcal {E}}_{s}}_{0,1})\\&=0. \end{aligned}$$

Conversely, let $\delta =\Psi \delta$ and $\vartheta _{n}\in {\mathscr {O}}(\vartheta )$ with $\vartheta _{n}\rightarrow \delta$.

Then

$$\begin{aligned} {\mathscr {G}}(\delta )={\mathcal {E}}_{s}(\delta ,\Psi \delta )&=0\\&\le \liminf _{n\rightarrow \infty }{\mathscr {G}}(\vartheta _{n})\\&={\mathcal {E}}_{s}(\Psi ^{n}\vartheta _{0},\Psi ^{n+1}\vartheta _{0}). \end{aligned}$$

This completes the proof. $\square$

## An RLC-electric circuit problem via fixed-point method

Most natural events can be mathematically explained, which typically results in an analysis of the challenges in terms of nonlinear differential equations. Being able to demonstrate that numerous traditional hypotheses from various scientific disciplines could be expressed in the context of linear differential equations demonstrates that, in quite a large number of essential instances, the mathematical formula in question can be linearized without losing any of its key components. However, to clarify and anticipate the observed behavior, some phenomena cannot be explained by linearizing the equations that characterize them. In these cases, a solution to the relevant nonlinear differential equations must be found. The study of nonlinear differential equations is becoming more and more important with the various subfields within multidisciplinary domains.

There has been a global interest in creating analytical techniques to solve nonlinear differential equations. In recent years, a number of literature works that provide an overview of the numerous approaches used to solve nonlinear differential equations have been established (see^28–31^). There aren’t many approaches developed for solving nonlinear problems that occur in real-world situations, according to a review of the literature on nonlinear differential equation solutions. The presented paper aims to use our main result of relevance in electrical circuits and to propose a method of solving nonlinear differential equations that is centered on the theory of nonlinear integral equations.

An RLC circuit typically refers to a circuit composed of a resistor (R), inductor (L), and capacitor (C), where the dynamics are governed by linear differential equations. However, when nonlinearities are introduced, such as nonlinear components or nonlinear behavior in the circuit elements, the analysis becomes more complex. In the case of nonlinear integral equations with Green’s functions, the analysis likely involves studying the response of the circuit to time-varying inputs or initial conditions, where the behavior of the circuit elements may not be adequately described by linear models. Green’s functions are useful for solving integral equations and can provide insight into the behavior of the system. Nonlinear integral equations can arise in various contexts within electrical engineering, such as in modeling nonlinear elements like diodes or transistors, or in describing complex behaviors like hysteresis or saturation effects in magnetic components.

The solution to such equations often requires numerical methods due to their complexity, and techniques like finite difference methods, finite element methods, or numerical integration may be employed to approximate the solutions.

### Example 3.1

Consider a simple series RLC circuit consisting of a resistor (R), an inductor (L), and a capacitor (C) connected in series to an AC voltage source. The general equation governing the behavior of this circuit is:

$$\begin{aligned} V(t)=I(t)R+L\frac{dI({\mathfrak {t}})}{d{\mathfrak {t}}}+\frac{1}{C}\int I({\mathfrak {t}})d{\mathfrak {t}}. \end{aligned}$$

Consider a specific example with $R=50$ ohms, $L=0.1$ Henry, $C= 100$ microfarads and $V(t)=10sin(100t)$ volts. Now, We want to find the current flowing through the circuit *I*(*t*).

We can solve this problem using differential equations, but let’s use a numerical method, such as the Euler method, for simplicity.

The Euler method is a basic numerical technique to approximate solutions of ordinary differential equations. Here’s how it works: Start with an initial condition:

(A) Start with an initial condition: $I(0)=0$ (assuming no initial current).
(B) Use the differential equation to find the rate of change of the current at each time step.
(C) Update the current using the rate of change and a small time step.

Now, perform this calculation for a small time step, say $\Delta {\mathfrak {t}}=0.01$ seconds, from ${\mathfrak {t}}=0$ to ${\mathfrak {t}}=2$ seconds.

**Figure 4:**
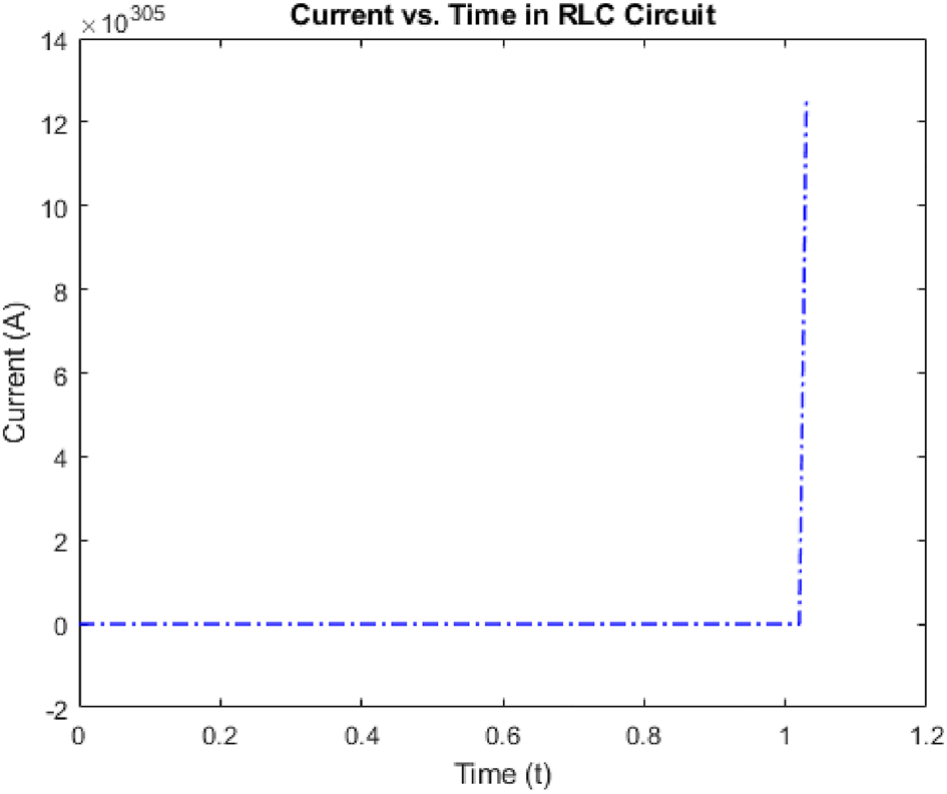
Current vs Time in an RLC Circuit.

Above Fig. 4 shows how the current in the circuit varies with time. We can observe the transient behavior as the circuit responds to the sinusoidal input voltage. Eventually, the current will stabilize to a sinusoidal waveform due to the balance between the inductive and capacitive reactance with the resistance.

### Existence of solution for the integral equation associated with an RLC electrical circuit equation

**Figure 5:**
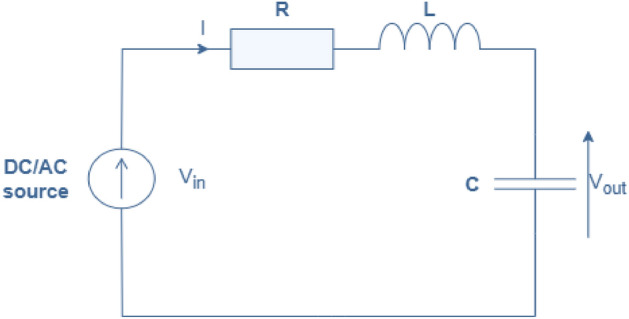
RLC-electrical circuit.

As a consequence of our results, the existence of solution for the integral equation associated with the electrical circuits equation problem described as below:

Consider an electrical circuit containing a resistor *R*, an inductor *L*, a capacitor *C* and total electro motive force $V({\mathfrak {t}})$ as shown in Fig. 5

By Kirchoff’s voltage law, we obtain

3.1 $$\begin{aligned} IR+\frac{q}{c}+L\frac{dI}{d{\mathfrak {t}}}=V({\mathfrak {t}}), \end{aligned}$$

where *I* is the current, and *q* is the charge.

Equation (3.1) can be written as a initial value problem

3.2 $$\begin{aligned} IR+\frac{q}{c}+L\frac{dI}{d{\mathfrak {t}}}=V({\mathfrak {t}}), \ \text {where} \ q(0)=0, \ \Big (\frac{dq}{d{\mathfrak {t}}}\Big )_{{\mathfrak {t}}=0}=0. \end{aligned}$$

The associated Green function is,

$$\begin{aligned} {\mathscr {G}}({\mathfrak {t}},\varsigma )= {\left\{ \begin{array}{ll} -\varsigma e^{\tau (\varsigma -{\mathfrak {t}})}, \ \text {if} \ 0\le \varsigma \le {\mathfrak {t}}\le 1, \\ -te^{\tau ({\mathfrak {t}}-\varsigma )}, \ \text {if} \ 0\le {\mathfrak {t}}\le \varsigma \le 1. \end{array}\right. } \end{aligned}$$

in which *R* and *L* are used to determine the constant $\tau >0$.

Assume that the collection that includes all continuous real-valued functions constructed on [0, 1] is ${\mathscr {M}}=({\mathcal {C}}[0,1],{\mathbb {R}})$. Let us define ${\mathcal {E}}_{s}:{\mathscr {M}}\times {\mathscr {M}}\rightarrow {\mathbb {R}}$ by ${\mathcal {E}}_{s}(\vartheta ({\mathfrak {t}}),\varrho ({\mathfrak {t}})) =\sup _{{\mathfrak {t}}\in [0,1]}\{|\vartheta ({\mathfrak {t}})-\varrho ({\mathfrak {t}})|^{2}e^{-|\tau {\mathfrak {t}}|}\}$.

Note that $({\mathscr {M}},{\mathcal {E}}_{s})$ is a complete ${\mathcal {E}}_{s}$-space with $b=2$ and $\gamma (\vartheta ({\mathfrak {t}}),\varrho ({\mathfrak {t}})) =e^{\vartheta ({\mathfrak {t}})+\varrho ({\mathfrak {t}})}$ for all $\vartheta ({\mathfrak {t}}),\varrho ({\mathfrak {t}})\in {\mathscr {M}}$, where $\gamma :{\mathscr {M}}\times {\mathscr {M}}\rightarrow [1,\infty )$.

The integral equation can be used to the problem mentioned above:

3.3 $$\begin{aligned} \vartheta ({\mathfrak {t}})=\int _{0}^{1}{\mathscr {G}}({\mathfrak {t}},\varsigma ) \kappa ({\mathfrak {t}},\vartheta (\varsigma ))d\varsigma , \ \text {where} \ {\mathfrak {t}}\in [0,1]. \end{aligned}$$

#### Theorem 3.2

Let ${\mathscr {M}}=({\mathcal {C}}[0,1],{\mathbb {R}})$ and let ${\mathscr {H}}:{\mathscr {M}}\rightarrow {\mathscr {M}}$ be the operator defined as

$$\begin{aligned} {\mathscr {H}}\vartheta ({\mathfrak {t}})=\int _{0}^{1}{\mathscr {G}}({\mathfrak {t}},\varsigma ) \kappa ({\mathfrak {t}},\vartheta (\varsigma ))d\varsigma , \ \text {for} \ {\mathfrak {t}},\varsigma \in [0,1], \end{aligned}$$

where $\kappa :[0,1]\times {\mathbb {R}}\rightarrow {\mathbb {R}}$ a continuous and non-decreasing function for all ${\mathfrak {t}}\in [0,1]$. Thus the problem (3.2) has a unique solution if the following assumptions hold.

1. $|\kappa ({\mathfrak {t}},\vartheta )-\kappa ({\mathfrak {t}},\varrho )|\le \tau ^{2}e^{-\tau (1-\frac{t}{2})}|\vartheta -\varrho |$ for all $\vartheta ,\varrho \in {\mathscr {M}}$ and ${\mathfrak {t}}\in [0,1]$ and $\tau >0$;
2. For all $$\vartheta ({\mathfrak {t}})\le \int _{0}^{1}{\mathscr {G}}({\mathfrak {t}},\varsigma ) \kappa ({\mathfrak {t}},\vartheta (\varsigma ))d\varsigma , \ \text {for all} \ {\mathfrak {t}}\in [0,1]$$.

#### Proof

Let $$\vartheta ({\mathfrak {t}}),\varrho ({\mathfrak {t}})\in ({\mathcal {C}}[0,1],{\mathbb {R}})$$. Consider,

$$\begin{aligned} |{\mathscr {H}}\vartheta ({\mathfrak {t}})-{\mathscr {H}}\varrho ({\mathfrak {t}})|&=\Big |\int _{0}^{1}{\mathscr {G}}({\mathfrak {t}},\varsigma )\kappa ({\mathfrak {t}}, \vartheta (\varsigma ))d\varsigma -\int _{0}^{1}{\mathscr {G}}({\mathfrak {t}},\varsigma ) \kappa ({\mathfrak {t}},\varrho (\varsigma ))d\varsigma \Big |\\&\le \int _{0}^{1}{\mathscr {G}}({\mathfrak {t}},\varsigma )|\kappa ({\mathfrak {t}},\vartheta (\varsigma )) -\kappa ({\mathfrak {t}},\varrho (\varsigma ))|d\varsigma \\&\le \int _{0}^{1}{\mathscr {G}}({\mathfrak {t}},\varsigma )\sup _{{\mathfrak {t}}\in [0,1]}| \kappa ({\mathfrak {t}},\vartheta ({\mathfrak {t}}))-\kappa ({\mathfrak {t}},\varrho ({\mathfrak {t}}))|d\varsigma \\&=\sup _{{\mathfrak {t}}\in [0,1]}|\kappa ({\mathfrak {t}},\vartheta ({\mathfrak {t}})) -\kappa ({\mathfrak {t}},\varrho ({\mathfrak {t}}))|\int _{0}^{1}{\mathscr {G}} ({\mathfrak {t}},\varsigma )d\varsigma \\&\le \tau ^{2}e^{-\tau (1-\frac{t}{2})}|\vartheta ({\mathfrak {t}}) -\varrho ({\mathfrak {t}})|\int _{0}^{1}{\mathscr {G}}({\mathfrak {t}},\varsigma )d\varsigma \\&\le e^{-\tau (1-\frac{t}{2})}|\vartheta ({\mathfrak {t}}) -\varrho ({\mathfrak {t}})|\big [1-2t\tau -e^{-t\tau }+\tau te^{\tau ({\mathfrak {t}}-1)}\big ]\\&\le e^{-\tau (1-\frac{t}{2})}|\vartheta ({\mathfrak {t}}) -\varrho ({\mathfrak {t}})|e^{-|\tau {\mathfrak {t}}|}\big [(1-2t\tau ) e^{\tau t}-1+\tau te^{\tau (2t-1)}\big ]\\&\le e^{-\tau (1-\frac{t}{2})}|\vartheta ({\mathfrak {t}}) -\varrho ({\mathfrak {t}})|e^{-\big |\frac{\tau t}{2}\big |}, \\&\ \ \ \ \ \ \ \ \ \ \ \ \ \ \ \ \ \ \ \ \ \ \text {as} \ \big [(1-2t\tau )e^{\tau t}-1+\tau te^{\tau (2t-1)}\big ]\le 1\\&=e^{-\tau (1-\frac{t}{2})}e^{-\big |\frac{\tau t}{2}\big |}| \vartheta ({\mathfrak {t}})-\varrho ({\mathfrak {t}})|, \end{aligned}$$

which yields that,

$$\begin{aligned} |{\mathscr {H}}\vartheta ({\mathfrak {t}})-{\mathscr {H}}\varrho ({\mathfrak {t}})|e^{-\big |\frac{\tau t}{2}\big |}\le e^{-\tau }e^{\big |\frac{-\tau t}{2}\big |}|\vartheta ({\mathfrak {t}})-\varrho ({\mathfrak {t}})|. \\ |{\mathscr {H}}\vartheta ({\mathfrak {t}})-{\mathscr {H}}\varrho ({\mathfrak {t}})|^{2}e^{-|\tau {\mathfrak {t}}|}\le e^{-2\tau }e^{-|\tau {\mathfrak {t}}| }|\vartheta ({\mathfrak {t}})-\varrho ({\mathfrak {t}})|^{2}. \\ \sup _{{\mathfrak {t}}\in [0,1]}|{\mathscr {H}}\vartheta ({\mathfrak {t}})-{\mathscr {H}}\varrho ({\mathfrak {t}})|^{2}e^{-|\tau {\mathfrak {t}}|}\le e^{-2\tau }\sup _{{\mathfrak {t}}\in [0,1]}|\vartheta ({\mathfrak {t}})-\varrho ({\mathfrak {t}})|^{2}e^{-|\tau {\mathfrak {t}}| }. \\ \Rightarrow {\mathcal {E}}_{s}({\mathscr {H}}\vartheta ({\mathfrak {t}}),{\mathscr {H}}\varrho ({\mathfrak {t}}))\le \frac{1}{e^{2\tau }}{\mathcal {E}}_{s}(\vartheta ({\mathfrak {t}}),\varrho ({\mathfrak {t}})) \\ \Rightarrow {\mathcal {E}}_{s}({\mathscr {H}}\vartheta ({\mathfrak {t}}),{\mathscr {H}}\varrho ({\mathfrak {t}}))\le \rho {\mathcal {E}}_{s}(\vartheta ({\mathfrak {t}}),\varrho ({\mathfrak {t}})). \end{aligned}$$

Therefore all the conditions of Theorem.2.1 satisfied, ${\mathscr {H}}$ has a fixed point. Consequently the differential equation arising in an RLC-electric circuit (3.2) guarantees the existence and uniqueness of the solution. $\square$

### Existence of solution for the integral equation associated with an RLC electrical circuit equation with a nonlinear element

Consider an RLC circuit with a nonlinear element, such as a diode. The diode’s current-voltage characteristic is typically described by a nonlinear equation, such as the Shockley diode equation.

The Shockley diode equation relates the current (I) through a diode to the voltage (V) across it and is given by:

$$\begin{aligned} I=I_{s}\Big (e^{\frac{V}{nV_{{\mathfrak {t}}}}}-1\Big ); \end{aligned}$$

where, *I* is the diode current, $I_{s}$ is the reverse saturation current, *V* is the voltage across the diode, *n* is the ideality factor, and $V_{{\mathfrak {t}}}$ is the thermal voltage, approximately 26*mV* at room temperature.

Now, let’s consider an RLC circuit with a voltage source $V_{s}({\mathfrak {t}})$, a resistor *R*, an inductor *L*, a capacitor *C*, and a diode *D* connected in series. The voltage across the diode $V_{D}({\mathfrak {t}})$ can be expressed as:

$$\begin{aligned} V_{D}({\mathfrak {t}})=V_{s}({\mathfrak {t}})-I(R)R-L\frac{dI}{d{\mathfrak {t}}}. \end{aligned}$$

The current-voltage relation for the diode gives us:

$$\begin{aligned} I(R)=I_{s}\Big (e^{\frac{V_{D}({\mathfrak {t}})}{nV_{{\mathfrak {t}}}}}-1\Big ). \end{aligned}$$

Substituting this expression for *I*(*R*) into the equation for $V_{D}({\mathfrak {t}})$, which yields,

$$\begin{aligned} V_{D}({\mathfrak {t}})=V_{s}({\mathfrak {t}})-I_{s}\Big (e^{\frac{V_{D}({\mathfrak {t}})}{nV_{{\mathfrak {t}}}}}-1\Big )R-L\frac{dI}{d{\mathfrak {t}}}. \end{aligned}$$

By Faraday’s law of electromagnetic induction, we know that the voltage across an inductor is given by the rate of change of current with respect to time multiplied by the inductance.

$$\begin{aligned} i.e., \ V_{L}({\mathfrak {t}})=L\frac{dI}{d{\mathfrak {t}}}. \end{aligned}$$

Therefore, we can rewrite $L\frac{dI}{d{\mathfrak {t}}}$ as $V_{L}({\mathfrak {t}})$, the voltage across the inductor:

$$\begin{aligned} V_{D}({\mathfrak {t}})=V_{s}({\mathfrak {t}})-I_{s}\Big (e^{\frac{V_{D}({\mathfrak {t}})}{nV_{{\mathfrak {t}}}}}-1\Big )R-V_{L}({\mathfrak {t}}). \end{aligned}$$

Now, the problem is to solve for $V_{D}({\mathfrak {t}})$ in terms of an integral equation. We can rewrite the equation in integral form by expressing $V_{L}({\mathfrak {t}})$ as an integral operator:

3.4 $$\begin{aligned} V_{D}({\mathfrak {t}})=V_{s}({\mathfrak {t}})-I_{s}\Big (e^{\frac{V_{D}({\mathfrak {t}})}{nV_{{\mathfrak {t}}}}}-1\Big )R-\int _{0}^{t}\frac{V_{D}(\tau )}{L}d\tau . \end{aligned}$$

This integral equation represents the voltage across the diode in terms of an integral of its own voltage over time, along with the input voltage $V_{s}({\mathfrak {t}})$ and the nonlinear term involving the diode current.

Let ${\mathscr {M}}$ be the space of continuous function on a closed interval [0, *T*]. Let us define ${\mathcal {E}}_{s}:{\mathscr {M}}\times {\mathscr {M}}\rightarrow {\mathbb {R}}$ by ${\mathcal {E}}_{s}(\vartheta ({\mathfrak {t}}),\varrho ({\mathfrak {t}})) =\sup _{{\mathfrak {t}}\in [0,T]}\{|\vartheta ({\mathfrak {t}})-\varrho ({\mathfrak {t}})|\}$.

Note that $({\mathscr {M}},{\mathcal {E}}_{s})$ is a complete ${\mathcal {E}}_{s}$-space with $b=2$ and $\gamma (\vartheta ({\mathfrak {t}}),\varrho ({\mathfrak {t}})) =e^{\vartheta ({\mathfrak {t}})+\varrho ({\mathfrak {t}})}$ for all $\vartheta ({\mathfrak {t}}),\varrho ({\mathfrak {t}})\in {\mathscr {M}}$, where $\gamma :{\mathscr {M}}\times {\mathscr {M}}\rightarrow [1,\infty )$.

#### Theorem 3.3

Let ${\mathscr {H}}:{\mathscr {M}}\rightarrow {\mathscr {M}}$ be the operator defined as

3.5 $$\begin{aligned} {\mathscr {H}}V_{D}({\mathfrak {t}})=V_{s}({\mathfrak {t}})-I_{s}\Big (e^{\frac{V_{D} ({\mathfrak {t}})}{nV_{{\mathfrak {t}}}}}-1\Big )R-\int _{0}^{t}\frac{V_{D}(\tau )}{L}d\tau . \end{aligned}$$

Then the Eq. (3.5) admits a unique solution if the following assumptions hold:

- If we choose $\delta$ such that, $|V_{D}-V^{\prime }_{D}|<\delta$;
- $$e^{\frac{\delta }{nV_{{\mathfrak {t}}}}}-1<\frac{1-\frac{T}{L}}{I_{s}R}$$.

#### Proof

Let $\vartheta ({\mathfrak {t}}),\varrho ({\mathfrak {t}})\in {\mathscr {M}}$. Now consider

$$\begin{aligned} |{\mathscr {H}}\vartheta ({\mathfrak {t}})-{\mathscr {H}}\varrho ({\mathfrak {t}})|&=|{\mathscr {H}}(V_{D})-{\mathscr {H}}(V^{\prime }_{D})| \\&=\sup _{{\mathfrak {t}}\in [0,T]}|{\mathscr {H}}(V_{D})({\mathfrak {t}}) -{\mathscr {H}}(V^{\prime }_{D})({\mathfrak {t}})|\\&=\sup _{{\mathfrak {t}}\in [0,T]}\Bigg |V_{s}({\mathfrak {t}})-I_{s} \Big (e^{\frac{V_{D}({\mathfrak {t}})}{nV_{{\mathfrak {t}}}}}-1\Big )R -\int _{0}^{t}\frac{V_{D}(\tau )}{L}d\tau -\Big (V_{s}({\mathfrak {t}}) -I_{s}\Big (e^{\frac{V^{\prime }_{D}({\mathfrak {t}})}{nV_{{\mathfrak {t}}}}} -1\Big )R-\int _{0}^{t}\frac{V^{\prime }_{D}(\tau )}{L}d\tau \Big )\Bigg |\\&=\sup _{{\mathfrak {t}}\in [0,T]}\Bigg |I_{s}\Big (e^{\frac{V_{D} ({\mathfrak {t}})}{nV_{{\mathfrak {t}}}}}-e^{\frac{V^{\prime }_{D}({\mathfrak {t}})}{nV_{{\mathfrak {t}}}}}\Big )R-\int _{0}^{t}\frac{V_{D}(\tau )-V^{\prime }_{D}(\tau )}{L}d\tau \Bigg |\\&\le \sup _{{\mathfrak {t}}\in [0,T]}\Bigg |I_{s}R\bigg |e^ {\frac{V_{D}({\mathfrak {t}})}{nV_{{\mathfrak {t}}}}}-e^{\frac{V^{\prime }_{D} ({\mathfrak {t}})}{nV_{{\mathfrak {t}}}}}\bigg |+\bigg |\int _{0}^{t}\frac{V_{D}(\tau ) -V^{\prime }_{D}(\tau )}{L}d\tau \bigg |\Bigg |\\&\le \sup _{{\mathfrak {t}}\in [0,T]}\Bigg |I_{s}R\Big |e^{\frac{\big |V_{D} ({\mathfrak {t}})-V^{\prime }_{D}({\mathfrak {t}})\big |}{nV_{{\mathfrak {t}}}}} -1\Big |+\frac{1}{L}\bigg |\int _{0}^{t}V_{D}(\tau )-V^{\prime }_{D}(\tau )d\tau \bigg |\Bigg |\\&\le I_{s}R\Bigg (e^{\frac{\sup _{{\mathfrak {t}}\in [0,T]}\big |V_{D} ({\mathfrak {t}})-V^{\prime }_{D}({\mathfrak {t}})\big |}{nV_{{\mathfrak {t}}}}}-1\Bigg ) +\frac{1}{L}|V_{D}-V^{\prime }_{D}|\int _{0}^{T}d\tau \\&= I_{s}R\Bigg (e^{\frac{\big |V_{D}-V^{\prime }_{D}\big |}{nV_{{\mathfrak {t}}}}}-1\Bigg )+\frac{T}{L}|V_{D}-V^{\prime }_{D}|\\&\le I_{s}R\Bigg (e^{\frac{\big |V_{D}-V^{\prime }_{D}\big |}{nV_{{\mathfrak {t}}}}}-1\Bigg )+\frac{T}{L}|V_{D}-V^{\prime }_{D}|\\&< \Bigg (I_{s}R\Bigg (e^{\frac{\big |V_{D}-V^{\prime }_{D}\big |}{nV_{{\mathfrak {t}}}}}-1\Bigg )+\frac{T}{L}\Bigg )|V_{D}-V^{\prime }_{D}|\\ \end{aligned}$$

Now, let’s define $k(=\frac{\eta }{b})$ such that:

$$\begin{aligned} k=I_{s}R\Bigg (e^{\frac{\big |V_{D}-V^{\prime }_{D}\big |}{nV_{{\mathfrak {t}}}}}-1\Bigg )+\frac{T}{L}. \end{aligned}$$

For $k<1$, we need,

$$\begin{aligned} I_{s}R\Bigg (e^{\frac{\big |V_{D}-V^{\prime }_{D}\big |}{nV_{{\mathfrak {t}}}}}-1\Bigg )+\frac{T}{L}<1 \\ \Rightarrow I_{s}R\Bigg (e^{\frac{\big |V_{D}-V^{\prime }_{D}\big |}{nV_ {{\mathfrak {t}}}}}-1\Bigg )<1-\frac{T}{L} \\ \Rightarrow e^{\frac{\big |V_{D}-V^{\prime }_{D}\big |}{nV_{{\mathfrak {t}}}}} -1<\frac{1-\frac{T}{L}}{I_{s}R}. \end{aligned}$$

Now if $|V_{D}-V^{\prime }_{D}|<\delta$, where $\delta$ is such that $e^{\frac{\delta }{nV_{{\mathfrak {t}}}}}-1<\frac{1-\frac{T}{L}}{I_{s}R}$, then $k<1$, and thus ${\mathscr {H}}$ satisfies Eq. (2.1). Therefore, if we choose $\delta$ such that $e^{\frac{\delta }{nV_{{\mathfrak {t}}}}}-1<\frac{1-\frac{T}{L}}{I_{s}R}$, then ${\mathscr {H}}$ satisfies Eq. (2.1), and Theorem.2.1 guarantees the existence and uniqueness of the solution to the integral equation (3.5) associated with an RLC electrical circuit equation with a nonlinear elements $\square$

## Open questions

- What are the additional conditions required in order to prove the existence of a solution and estimation of distortion of Chua’s circuit^32^ for the above-obtained results in ${\mathcal {E}}_{s}$-space?
- Prove or disprove Lagrangian for Chua’s circuit through distance space.

## Data Availability

The data sets used and/or analyzed during the current study available from the corresponding author on reasonable request.
